# Public health response to large influx of asylum seekers: implementation and timing of infectious disease screening

**DOI:** 10.1186/s12889-018-6038-9

**Published:** 2018-09-24

**Authors:** Paula Tiittala, Karolina Tuomisto, Taneli Puumalainen, Outi Lyytikäinen, Jukka Ollgren, Olli Snellman, Otto Helve

**Affiliations:** 1 Doctoral Programme for Population Health, University of Helsinki, Helsinki, Finland; 2 Department of Health Security, Infectious Disease Control and Vaccinations Unit, National Institute for Health and Welfare, P.O. Box 30, 00271 Helsinki, Finland; 3 Finnish Immigration Service, Helsinki, Finland

**Keywords:** Asylum seeker, Screening, Infectious diseases, Public health response, Preparedness

## Abstract

**Background:**

Infectious disease screening of migrants at increased risk is a feature of national infection prevention and control measures. Asylum seekers in Finland are offered screening of tuberculosis (TB), hepatitis B, human immunodeficiency virus infection (HIV) and syphilis based on individual risk assessment. We aimed to evaluate the public health response to a large influx of asylum seekers to Finland in 2015–2016 with respect to national guidelines on initial health services and infectious disease screening.

**Methods:**

We used immigration and healthcare procurement data for all 38,134 asylum seekers to Finland during 2015–2016 to assess the implementation, timing and yields of infectious disease screening.

**Results:**

The coverage of pulmonary TB screening was 71.6% [95% CI 71.1–72.0%] and that of hepatitis B, HIV or syphilis 60.6% [60.1–61.1%] among those eligible for screening. The estimated average delay from arrival to pulmonary TB screening was 74 days for adults and 43 days for children. Delay to hepatitis B, HIV and syphilis screening was 91 days for adults and 47 days for children. The seroprevalence of hepatitis B surface antigen positivity was 1.4% [95% CI 1.3–1.6%], HIV 0.3% [95% CI 0.1–0.4%] and *Treponema pallidum* specific antibodies 1.0% [95% CI 0.8–1.1%]. Data did not allow assessment of yields of pulmonary TB screening.

**Conclusions:**

Up to one third  of asylum seekers were not reached by screening and screenings were delayed from target timeframes. Children, as a vulnerable population, were screened earlier than adults. To ensure higher screening coverage, infectious disease risks should be reassessed and screening completed at contacts to healthcare during the post-asylum phase of integration. The large influx of asylum seekers to Finland in 2015–2016 tested the country’s public health preparedness. After action reviews of the public health response to the large migrant influx such as screening implementation can be used for evidence-based improvement of public health preparedness and guidelines for initial health services and infectious disease screening.

## Background

Migrants may have an increased risk for certain infectious diseases, including tuberculosis (TB), hepatitis and human immunodeficiency virus infection (HIV), depending on factors such as the prevalence of the disease, the living conditions and potential healthcare service disruptions in their country of origin and during transit, as well as individual risk behaviours [1]. European member states of World Health Organization (WHO) have committed themselves to ensuring the necessary capacities to address communicable diseases among migrant populations [2]. Several European countries, including Finland, have adopted screening protocols for refugees and asylum seekers [3–5], with substantial variation between countries and regions. Screening of infectious diseases aims to detect a disease at an early stage, and thus to protecting both the individual and the population [6]. Screening should be acceptable, cost-effective and follow-up services made available. [7]

An asylum seeker is a person seeking international protection from a foreign country and awaiting the decision on their application for refugee status [8].

In the autumn of 2015, Europe experienced a large-scale arrival of asylum seekers, mainly from Middle Eastern countries and the Horn of Africa [9, 10]. The nearly ten-fold increase in asylum seekers to Finland was among the highest in Europe [11, 12]. This “migration crisis” triggered the largest domestic relief operation and public health response since World War II [13].

The public health response to a large influx of asylum seekers, and specifically screening for infectious diseases, is an important dimension of national public health emergency preparedness [14]. The public health response determines the health and human rights outcomes for migrants, as well as the host population [9]. Ensuring the highest attainable health for migrants requires an effective health system response and removal of barriers to healthcare [15]. Within the WHO Health 2020 framework, Public Health Aspects of Migration in Europe (PHAME) project [9] and International Health Regulations (IHR 2005), countries of the WHO Europe region are assessing and evaluating their capacities to respond to the healthcare needs of a large influx of asylum seekers.

While there are several reports on the screening yields of infectious diseases among newly-arrived asylum seekers to Europe [16–20], only a few [21, 22] have focused on describing the implementation of the public health response. As dimensions of the public health response, we aimed to evaluate the implementation of the national guidelines, including screening coverage, timing, assessment of vulnerability and screening yields, during the large influx of asylum seekers to Finland in 2015–2016.

## Methods

### Organisation of asylum seekers’ healthcare in Finland

The Finnish Immigration Service (Migri) falls under the Ministry of the Interior and is in charge of the asylum process in Finland. The Migri is also responsible for organizing sufficient reception center capacity and coordination, planning and supervision of the practical aspects of healthcare for asylum seekers in Finland.

All registered asylum seekers in Finland have a designated reception centre which organises their reception services including housing, board, and health and social services. Private for-profit and not-for-profit organisations are amongst the providers of reception services. Typically, asylum seekers are first accommodated in so-called transit centres for a period of days to weeks before they are assigned to a reception centre for longer term settlement, until their asylum claim has been processed. Asylum seekers who live in private housing are also assigned to receive services from a particular reception centre.

According to the Act on the Reception of Asylum Seekers (746/2011), adult asylum seekers in Finland are entitled to urgent and necessary healthcare. Asylum-seeking children under 18 years of age are entitled to the same level of care as permanent residents. Registered nurses who work in the transit and reception centres coordinate the organisation of health services for asylum seekers. The nurses have a public health, midwifery or specialised care background. Nurses initiate the voluntary health examinations at the centres and provide primary level nursing; however, other healthcare services, including the actual screenings, are purchased from public or private health services.

### Infectious disease screening among asylum seekers

In Finland, the legal obligation for the infectious disease control lies with the 311 municipalities, 20 hospital districts and 6 Regional State Administrative Agencies. The Ministry for Social Affairs and Health (STM) is responsible for the overall management and takes the lead in public health emergencies.

According to STM guidelines [23], asylum seekers and refugees in Finland are offered multiphasic selective screening of TB, hepatitis B, HIV and syphilis based on an individual risk assessment (Table 1). A symptom-based questionnaire is used to identify those with possible symptoms of active TB. Pulmonary TB is screened with a chest X-ray (CXR) from two projections right after the health examination, which is arranged within 2 weeks of arrival. Children under 7 years of age who have not received a Bacillus Calmette-Guérin vaccine are also screened for latent and extra-pulmonary TB. The screening of hepatitis B, HIV, and syphilis is arranged within 3 months of arrival. Children under 16 years of age are screened for intestinal protozoa and helminth eggs within 3 months of arrival. Participation in screenings is voluntary and a written informed consent is obtained from participants or their legal representatives.

**Table 1:** National infectious disease screening recommendations for asylum seekers

Infectious disease	Target population	Screening indication	Screening test
Pulmonary tuberculosis	All	Tuberculosis (TB) incidence 50/100000 or above in the country of origin, or originating from conflict areas, or having lived in camp settings, or close contacts to TB patients, or symptoms of TB	Chest X-ray from two^a^ projections (CXR)
Latent extra-pulmonary tuberculosis	BCG-unvaccinated children under 7 years of age	Tuberculosis (TB) incidence 50/100000 or above in the country of origin, or originating from conflict areas, or having lived in camp settings, or close contacts to TB, or symptoms of TB	Interferon Gamma Realease Assay (IGRA)
Hepatitis B	All	HBsAg prevalence above 2% in the country of origin or transit	Serum Hepatitis B surface antigen (HBsAg)
HIV	All	HIV prevalence above 1% in the country of origin or transit, or specific risk behavior such as injecting drug use, sex between men, incarceration, commercial sex work, or an individuals own request	Serum Human Immunodeficiency Virus antigen and antibodies (HIVAgAb)
Syphilis	All	If HBsAG or HIVAgAb screening is performed	Serum *Treponema pallidum* antibodies (anti-Trpa)
Intestinal parasites	Children under 16 years of age	Country of origin or transit in South-East Asia, India or Sub-Saharan Africa	Direct microscopy of fecal parasites

^a^With the exception for asymptomatic pregnant women, CXR is recommended to be perfomed after 36 gestational weeks and from anterio-posterior projection only

The reception centre nurse assesses each individual’s infectious disease risk based on relevant disease epidemiology in the countries of origin and transit, as well as individual medical history and risk behaviours. Professional interpreters are used if necessary. Priority is given to individuals in vulnerable situations or presenting with symptoms; children, pregnant women, and disabled persons are referred to a doctor’s appointment. The National Institute for Health and Welfare publishes guidance documents to support the decision making and risk assessment processes, including information on the global infectious disease prevalence [24].

### Study design

We performed a cross-sectional retrospective register-based study to by comparing data from two different sources: the immigration register and the healthcare procurement register. We were not able to link the two data sources on an individual level and hence the data were analysed in parallel. The immigration register included monthly information on the nationalities and age groups of asylum seekers. Healthcare procurement register collected monthly information on the number and kind of procurements for different age groups. Migri had contracted out the infectious disease screenings of asylum seekers to two national private service providers, who the reception centres purchase the screening services from. Since asylum seekers are generally not assigned a personal identification number, which would facilitate the use of data from the patient information systems, and since the reception centres do not consistently report screening findings, procurement data were the only feasible source with which to assess screening coverage and yields.

All asylum seekers to Finland in 2015–2016 were included in the analyses. Individuals with missing information on nationality or unknown nationality were excluded. Data for the study were collected from January 1st 2015 till December 31st 2016. Results were stratified by age. Individuals under 18 year of age were considered children.

### Outcomes

Screening coverage was calculated as the number of screenings performed (numerator) against the number of asylum seekers eligible for screening (denominator). Eligibility for screening of pulmonary TB and syphilis was determined according to the national guidelines after assuming the country of nationality as the country of origin [23, 24]. Syphilis screening was used as an indicator for any blood screening performed since, according to national guidelines, syphilis screening is recommended whenever either hepatitis B or HIV is screened.

**Fig. 1:**
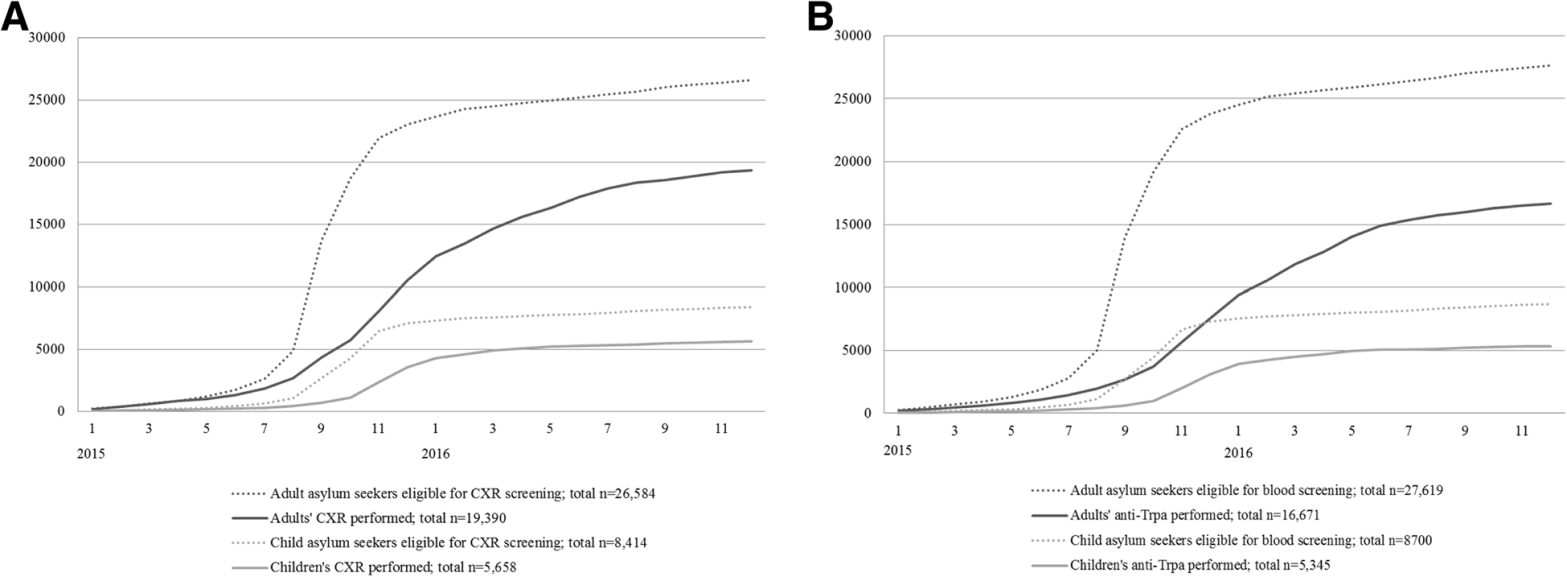
Cumulative absolute number of chest X-ray (**a**) and anti-Trpa screenings (**b**) performed in 2015–2016 in comparison to cumulative absolute number of arrival of screening eligible asylum seeking children and adults

**Fig. 2:**
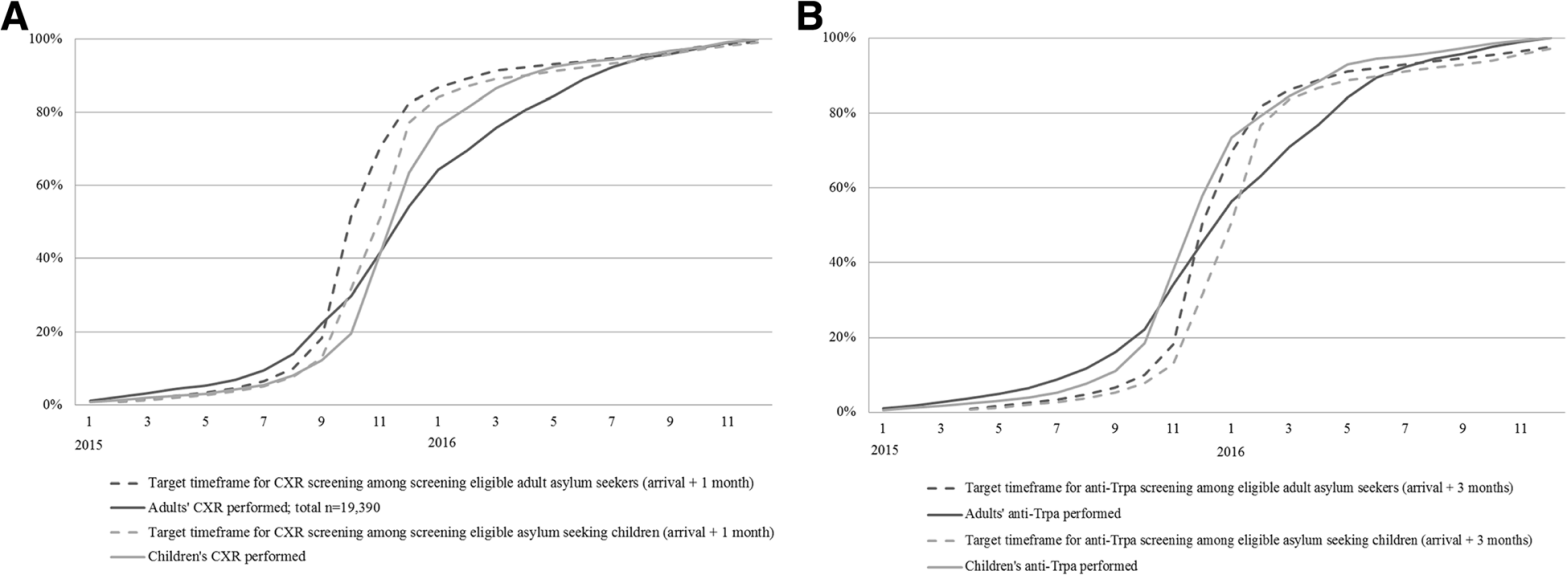
Cumulative relative proportion of performed chest X-ray (**a**) and anti-Trpa screenings (**b**) out of all screenings performed by month in 2015–2016 against the target timeframes for screenings for children and adults

Timing of screening was assessed by comparing the dates of arrival to the dates of screening performed stratified by age group. As we were not able to link the data at individual level, we calculated the median and average dates of arrival and screening at population level. The delay to screening was calculated as the difference between average dates of arrival and screening. Dynamics of achieving the screening coverage was depicted visually by plotting the cumulative absolute number of CXR and syphilis serology screenings performed for adults and children against the number of new asylum seekers to Finland during 2015–2016 from screening-eligible countries (Fig. 1). To portray the delays of performed screenings against the target timeframes as per national guidelines, we plotted the cumulative relative proportion of screenings performed out of all screenings performed against the cumulative relative proportion of asylum seekers eligible for screening adjusted with the target timeframes for screening (Fig. 2). Screenings were considered delayed when the plot for cumulative proportion of screenings performed took place later than the plot for target timeframe for screenings set in the national guidelines. The dates were assumed as 15th of each month. A target timeframe for screening was set at 1 month after arrival for CXR screening and at 3 months after arrival for syphilis screening as per national guidelines [23].

Screening yields were presented as the proportion of seropositive findings (numerator) out of all screenings performed (denominator) stratified by age groups.

### Laboratory diagnostics

Serum hepatitis B surface antigen (HBsAg) and serum HIV antigen and antibodies (HIVAg/Ab) were determined using chemiluminescence microparticle immunoassays (CMIA) (ARCHITECT® i1000SR or i2000SR HIV Ag/Ab Combo and ARCHITECT® i1000SR or i2000SR HBsAg qualitative respectively, Abbot, Chicago, Illinois, USA). HIVAg/Ab positive findings were confirmed with an immunoblot assay in the reference laboratory (TYKSLAB, Turku, Finland or HUSLAB, Helsinki, Finland). Serum Treponema pallidum antibodies (anti-Trpa) were determined using CMIA (IMMULITE® 2000XPi, Siemens, Munchen, Germany or ARCHITECT® i2000SR, Abbot). The blood lymphocyte reactivity to Mycobacterium TB antigens was determined using an Interferon Gamma Release Assay (IGRA) (QuantiFERON-TB Gold Plus, Qiagen, Hilden, Germany). Positive cases were referred to a doctor’s consultation.

### Statistical considerations

The data analysis was performed using Microsoft Excel 2010 (Microsoft, Redmont, Washington, USA). The confidence intervals (CI) were calculated according to Wald. Pearson’s Chi-squared was used to test the null hypothesis and *p*-values of 0.05 or below was considered statistically significant. Median and average dates of arrival and screening are presented. Average delay to screening was considered as the difference in means of arrival and screening after assuming that E(x-y) = Ex - Ey where E is the expected value and a linear operator. The study was endorsed by the Research Ethical Committee of the National Institute for Health and Welfare (§758, 16.3.2017).

## Results

A total of 38,134 individuals sought asylum in Finland during 2015–2016. Due to missing information regarding their nationality, 520 (1.4%) individuals were excluded from analyses. Accurate information on the number of asylum seeking children was available; all other individuals, including the 190 (0.5%) individuals with missing information on age, were assumed to be adults. Of the 37,614 asylum seekers included in the final study population, one in four (24.0%, *n* = 9031) of the applicants were under 18 years of age, 37.6% (*n* = 3400) of whom were unaccompanied by a guardian. The majority of applicants were young adult men in the age group of 18 to 34 years (Table 2).

**Table 2:** Basic characteristics of the asylum seekers to Finland in 2015–2016

Characteristic	2015; n (%), total *n* = 32,477	2016; n (%), total *n* = 5657	Total; n (%), total *n* = 38,134
Age at asylum appeal, years^a^			
0–13	4250 (13.1)	1419 (25.1)	5669 (14.9)
14–17	3402 (10.5)	338 (6.0)	3740 (9.8)
18–34	19,585 (60.3)	2812 (49.7)	22,397 (58.7)
35–64	4995 (15.4)	1029 (18.2)	6024 (15.8)
65 or above	78 (0.2)	36 (0.6)	114 (0.3)
Men^b^	26,424 (81.4)	3698 (65.4)	30,122 (79.0)
Origin^c^			
Iraq	20,484 (63.1)	1247 (22.0)	21,731 (57.0)
Afganistan	5214 (16.1)	757 (13.4)	5971 (15.7)
Somalia	1981 (6.1)	432 (7.6)	2413 (6.3)
Syria	877 (2.7)	602 (10.6)	1479 (3.9)
Other Middle East and North Africa	1235 (3.8)	813 (14.4)	2048 (5.4)
Europe	1060 (3.3)	203 (3.6)	1263 (3.3)
Sub-Saharan Africa	545 (1.7)	576 (10.2)	1121 (2.9)
Asia	311 (1.0)	493 (8.7)	804 (2.1)
Former Soviet Union Countries	373 (1.1)	351 (6.2)	724 (1.9)
Other	29 (0.1)	31 (0.5)	60 (0.2)

^a^For 190 individuals (0.5%), the age at immigration was missing. ^b^For 55 (0.1%) individuals, information on the sex was missing. ^c^Origin is based on nationality. Nationality was unknown for 383 (1.0%) individuals and 137 (0.4%) applicants had no nationality

### Prevalence of eligibility

Of the asylum seekers included in the study population, 91.8% [95% CI 91.5–92.0%] were eligible for TB screening and 95.2% [95.0–95.5%] for hepatitis B, HIV or syphilis. There were no significant differences in screening eligibility between children and adults for either CXR or blood screenings (*p* = 0.89 and 0.81, respectively).

### Coverage of screening

The overall coverage of CXR screening among eligible adults and children was 71.6% [95% CI 71.1–72.0%], with 25,048 examinations performed. CXR screening was performed for 19,390 adults and 5658 for children. The CXR coverage among adults was 72.9% [72.4–73.5%] and among children 67.2% [66.2–68.2%] (*p* < 0.01 between age groups). The overall coverage of syphilis screening was 60.6% [60.1–61.1%], with 22,016 tests performed for those eligible for screening. Anti-Trpa tests were performed for 16,671 adults and 5345 children. The coverage of syphilis screening was 60.4% [59.8–60.9%] among adults and 61.4% [60.4–62.5%] among children (*p* = 0.26 between age groups).

### Timing of screening

Median date of asylum appeal was September 2015 for adults and October 2015 for children. Median date of CXR screening was December 2015 for both adults and children. Median date anti-Trpa screening was January 2016 for adults and December 2015 for children. Comparison of the cumulative absolute numbers of arrivals to the absolute numbers of screenings performed shows how the screening coverage changed over time (Fig. 1). Cumulative absolute number of arrival of screening eligible asylum seeking children and adults plateaued by beginning of 2016 due to decreasing number of new applications (Fig. 1). The cumulative absolute number of CXR (Fig. 1a) and anti-Trpa screenings (Fig. 1b) performed in 2015–2016 plateaued later in May–July 2017.

The estimated average delay from arrival to CXR screening among those who were screened was 74 days for adults and 43 days for children. The average delay to syphilis screening was 91 days among screened adults and 47 days among screened children. Comparison of the cumulative relative proportion of performed screenings out of all screenings performed by month in 2015–2016 against target timeframes depicts the delays in screenings (Fig. 2). For adults, the CXR screenings were delayed vis-à-vis the target timeframe from September 2015 to October 2016 (Fig. 2a). For children, the CXR screenings began to lag in September 2015, but were already back on schedule by April 2016. Anti-Trpa screening of adults was delayed from November 2015 until July 2016, but children’s screenings remained on schedule throughout the follow-up period (Fig. 2b).

No differences in timing of CXR or syphilis screening among children were observed between the two service providers. However among adults, both CXR and syphilis screenings were performed later (median March 2016 and May 2016 respectively) by the provider serving the Helsinki metropolitan area in comparison to the provider serving the rest of Finland (median December 2015 for both CXR and syphilis screening respectively).

### Prevalence of infections

Based on the screening results, we estimated the prevalence of infectious diseases among the study population. The overall HBsAg prevalence was 1.4% [95% CI 1.3–1.6%]. HBsAg prevalence was significantly higher among adults (1.6%, 95% CI 1.4–1.8%, *n* = 274) than among children (0.8%, 95% CI 0.6–1.1%, *n* = 44) (*p* < 0.01). Ten HBsAg-positive cases were identified in children less than 15 years of age.

The overall HIV prevalence was 0.3% [95% CI 0.1–0.4%, *n* = 45] with no cases in children. The total prevalence of positive syphilis serology was 1.0% [95% CI 0.8–1.1%]. The prevalence of syphilis serology among adults (1.2%, 95% CI 1.0–1.4%, *n* = 199) was significantly higher than among children (0.2%, 95% CI 0.1–0.4%, *n* = 12) (*p* < 0.01). Four anti-Trpa positive cases were identified in children less than 15 years of age. Only one IGRA-positive case was identified in a child less than 7 years old, with the prevalence being 0.6% [95% CI 0–0.8%].

Comparison of prevalence rates between the two service providers and between different years indicated that HIV prevalence was significantly higher in data reported by the provider operating in the Helsinki metropolitan area compared to the provider serving the rest of the country (1.5 and 0.1% respectively, p < 0.01). Prevalence of HIV and anti-Trpa positivity was significantly lower in 2015 (0.2 and 0.7% respectively) as compared to 2016 (0.5 and 1.2% respectively, *p* < 0.05). We did not observe differences in reported prevalence rates of HBsAg, anti-Trpa nor IGRA between service providers nor in different years.

## Discussion

We described the public health response to the large influx of asylum seekers to Finland during 2015–2016 by evaluating the implementation and timing of infectious disease screenings using healthcare procurement data. As a contrast to other studies performed in healthcare settings [19, 20] or in selected regions of destination countries [16–20, 25–28], our study included an national asylum seeker population, which reinforces the external validity of our results. Due to the service providers’ financial interests, the healthcare procurement data are likely to be accurate, available and internally valid. In Finland, the asylum seekers’ health services were contracted out to only two national service providers, which facilitated the data collection.

### Screening coverage

Similar to findings from a recent systematic review on coverage of infectious disease screening among migrants [29], the screening coverage of pulmonary TB and hepatitis B, HIV and syphilis among asylum seekers to Finland during 2015–2016 remained modest. The large influx of asylum seekers to Finland during this time stretched the health system’s capacity, especially in the areas of most frequent border crossings and the transit centre locations – at the Northern border between Finland and Sweden (Tornio) and Helsinki metropolitan area. Facing this pressure, some sub-national health authorities decided to limit the scope or delay infectious disease screenings services for asylum seekers as opposed to national guidelines [30]. Therefore, the observed deficits in coverage can result from regional differences in screening practices.

The significantly higher CXR screening coverage for adults than children can be explained by the fact that in clinical practice, adults with suspicion of a lower respiratory tract infection are more likely to be referred to a diagnostic CXR than children. Moreover, to support this, blood screening coverage between children and adults did not differ. As a study limitation, procurement data do not differentiate the indication of a test, therefore, the total number of CXRs also includes diagnostic X-rays.

### Timing of the response

The monthly accumulation of the number of screening tests in comparison to the number of asylum applications from screening-eligible countries shows that the intensity of screening for pulmonary TB, hepatitis B, HIV and syphilis began to lag as the number of arriving asylum seekers increased exponentially in September 2015 (Fig. 1). The accumulation of both arrivals and screenings plateaued by May–July 2016. This stagnation in the cumulative number of new asylum applications is explained by the decreasing number of arrivals to Finland in spring 2016. However, the simultaneous plateau in cumulative number of screenings performed suggests structural reasons for suboptimal coverage.

Children arrived slightly later to Finland, compared to adults, but were screened earlier (Fig. 2). The average delay from arrival to CXR screening exceeded the timeframe set in the national guidelines (1 month) for both adults (74 days) and children (43 days) [23]. However, for children, the delay from arrival to blood screening (47 days) remained within the target timeframe (3 months). The average delay in screening was almost twice as long for adults than for children for both CXR and anti-Trpa screening. Considering the high number of individuals in our data, the result is likely to be highly significant although we are unable to tests the statistical significance. The observed prioritisation of children as a vulnerable population is in accordance with national guidelines [23], international agreements [9] and healthcare ethics. 

Among adults, CXR and blood screening were delayed especially in Helsinki metropolitan region which might reflect the impact of the sub-national guidelines [30]. However, we are not able to assess the average delay from arrival to screening between the two service providers due lacking information regarding the region in the immigration data.

In general, delays in the implementation of screening reflect the capacity of the healthcare system to respond to sudden changes, in this case, to a large increase in the demand for services. Considering that up to one third of asylum seekers were not reached by screening, the presented delays are likely to be underestimates. A delay in screening might result in longer periods of infectiveness, increased morbidity and possible transmission of the disease [1].

To best of our knowledge, the only other study to evaluate the delay from arrival to implementation of infectious disease screening during the European migrant crisis in 2015–2016 was reported from Sweden [21]. While in the Sweden a majority of the unaccompanied asylum seeker children had arrived by January 2015, 96% of the referrals were received before June 2016 suggesting a delay from arrival to screening of approximately 5 months.

### Infectious disease prevalence

The observed HBsAg seroprevalence of 1.4% was lower as compared to majority of previous studies among asylum seeker in Europe [17, 18, 20, 25–28, 31]. A hepatitis B seroprevalence study among adult asylum seekers from Middle-East and Horn of Africa in the Netherlands in 2016 concluded a lower HBsAg prevalence of approximately 1% as compared to our study [32]. Differences in seroprevalence rates are most likely due to differences in countries of origin and age distributions among the recently-arrived asylum seeker populations. In our sample, the proportion of asylum seekers from Sub-Saharan Africa and Asia shown to have highest risk for hepatitis B, was low [33]. On the other hand, Syrian migrants have been shown to have low HBsAg prevalence [19, 27], but Syrians represented only a clear minority in our study population. The observed HIV prevalence of 0.3% was lower or comparable to other studies among asylum seekers to Europe during the migrant crisis in 2015–2016 [16, 20, 25–28, 31].

Estimates of syphilis seroprevalence among asylum seekers in Europe in 2015–2016 are sparse and vary according diagnostic tests used. The 1.0% seroprevalence of anti-Trpa positivity in our study was higher or comparable as shown in previous studies with a similar population tested with the same diagnostic methods [16, 31]. Anti-Trpa positive cases were referred to a doctor’s consultation and further diagnostics were performed to determine whether a case represented active syphilis requiring treatment or whether positivity was due to a previously-treated infection and was thus an immunological scar. More information is needed to understand the socio-demographic risk factors and clinical features of the anti-Trpa positive cases. Having said this, the results on syphilis prevalence support the inclusion of anti-Trpa screening in the national guidelines.

The low prevalence of IGRA positivity among children less than 7 years of age not vaccinated with BCG can be explained by the selected population.

A higher HIV prevalence was reported by the provider serving the Helsinki metropolitan area which might result from heterogenous distribution of asylum seekers of different origins in Finland. From our data, we were unable to assess the specific location of testing and it’s influence on timing fo screening. Differences in HIV and anti-Trpa prevalence observed between 2015 and 2016 can be explained by differences in countries of origin among the asylum seekers (Table 2).

### Limitations

Using healthcare procurement data instead of individual level data resulted in an inability to evaluate the influence of socio-demographic background, previous patient history, or concurrency of infections. It was also not possible to assess regional differences or, on an individual level, screening coverage or screening delays. Additionally, we were unable to assess the screening yields of TB, since all suspected cases are referred to tertiary level care for further examinations. Prevalence estimations are affected by a selection bias as infection prevalence might differ between asylum seekers who participated in the screenings as opposed to those who did not.

Factors contributing to the possible underestimation of the screening coverage are three-fold. Firstly, some asylum seekers might have left the country before the screenings were performed. The average duration of the asylum process has been estimated to be between 6 and 24 months [34], hence, the number of aborted appeals prior to infectious disease screening and their contribution to the underestimation of the screening coverage is likely to be small.

Secondly, we obtained screening data from the two nationally-contracted healthcare providers, but some reception centres might have procured their services from third party providers. Because the contracted service providers can easily be reached from the vast majority of reception centres, the proportion of services procured from third party healthcare providers is likely to be small. Thirdly, we were unable to assess the acceptability of screening. However, in a recent systematic review among asylum seekers in Europe, acceptability of screening has been shown to be high [29].

There are also important structural delays to consider, from arrival to screening (up to 3 months) and from screening to financial reporting (up to 2 months). Due to these structural delays, our data include screenings performed for asylum seekers who have arrived in late 2014 and excludes screenings of those who arrived in late 2016. However, since the numbers of asylum applications in the second half of 2014 and 2016 were similar, the effect on our analysis is likely to be very small.

## Conclusions

Evaluations of screening programs should consider dimensions of validity, reliability, yield, cost, acceptance and availability of follow-up services [7]. Our study adds to the sparse evidence that the effectiveness of a screening program might be compromised in real world conditions especially in a time of a crisis [35, 36]. Barriers to achieving high coverage of infectious disease screening among asylum seekers should be recognized and addressed [29].

Debate on the infectious disease risks to public health has accompanied the migrant crisis in Europe. The debate is fuelled from one side with fear of spread of infectious diseases and doubts on the cost-effectiveness of screening on the other [37]. Austerity measures adopted by some countries in Europe restrict asylum seekers’ access to services and increased risks to infectious disease hazards [36]. Public health authorities have taken a clear stance on asylum seekers being vulnerable to infections themselves rather than generating a public health threat [37–39].

The large influx of asylum seekers to Finland in 2015–2016 put the health system under pressure; the implementation of infectious disease screening was delayed in comparison to the national guidelines and the overall screening coverage remained sub-optimal. Results demonstrate that asylum-seeking children, as a vulnerable population, were prioritised in healthcare. Recognising the modest screening coverage during the asylum process, completion of screening and infectious disease risks should be reassessed at contacts to healthcare during residency and integration to ensure a higher screening coverage.

Infectious disease screening is an important dimension of the public health response to a large influx of migrants. In the current global political, ecological and economic climate, continued migration is likely. However, in order to describe the effectiveness of the response as a whole, more information is needed on the resources, morbidity, usage of healthcare services, and of alternative response strategies. Infectious disease control measures should be ethically justified especially when targeting marginalized populations such as asylum seekers [6]. We need to draw lessons from the diverse responses of the European migrant crisis of 2015–2016 and develop evidence-based approaches to ensure better public health emergency preparedness in the future.

## Abbreviations

Anti-Trpa: *Treponema pallidum* antibodies
BCG: Bacillus Calmette-Guérin
CI: confidence interval
CXR: chest X-ray
HBsAg: hepatitis B surface antigen
HIV: human immunodefieciency virus infection
IGRA: Interferon Gamma Release Assay
IHR: international health regulations
IQR: inter-quartile range
Migri: Finnish Immigration Service
PHAME: Public Health Aspects of Migration in Europe
SD: standard deviation
TB: tuberculosis
WHO: World Health Organization
